# Supplementary material for: Identification of Carotenoids in Hairless Canary Seed and the Effect of Baking on Their Composition in Bread and Muffin Products
Source: Molecules. 2022 Feb 15;27(4):1307. doi: 10.3390/molecules27041307 (PMC8875352; doi:10.3390/molecules27041307)
Supplement: Supplementary file 1 [file molecules-27-01307-s001.zip › molecules-1594870-supplementary.pdf]

Supplementary Figure S1

Appearance of muffin and bread products. C, corn; HCS, hairless canary seed and W, wheat.

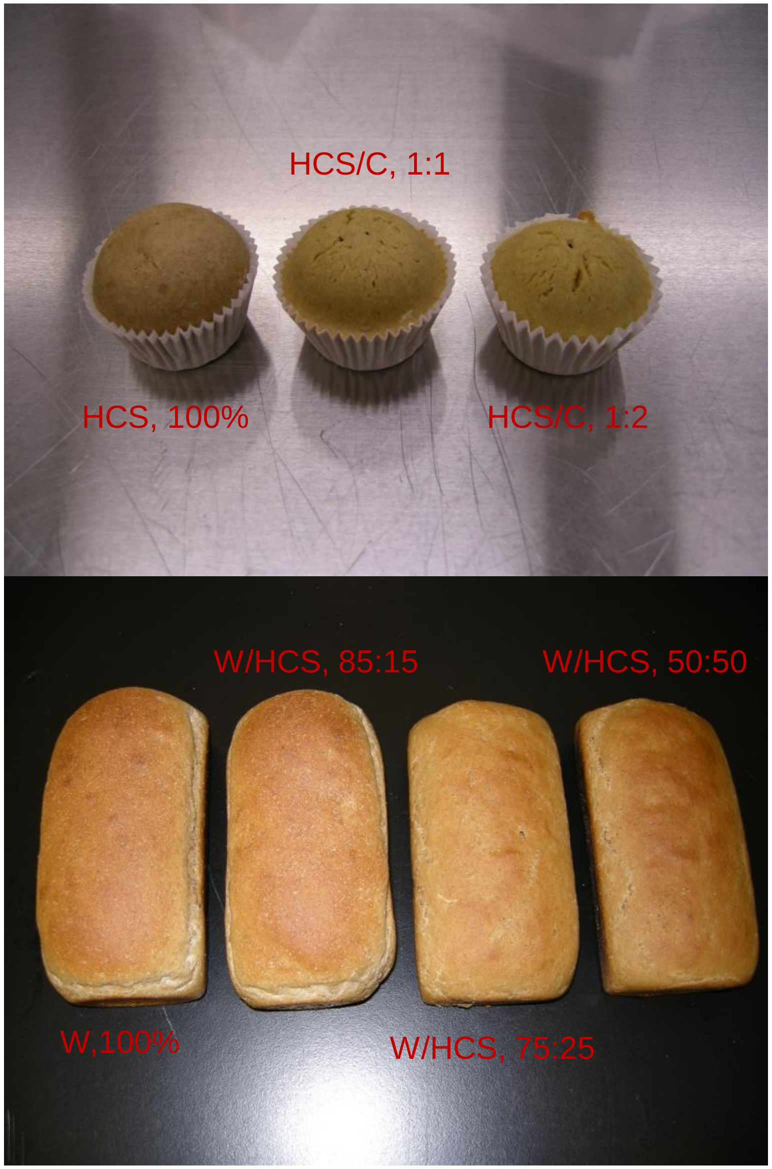

Supplementary Figure S2

MS spectra of (A) lutein-3-*O*-palmitate in ESI mode, (B) lutein-3’-*O*-palmitate in ESI mode (C) lutein-3-*O*-palmitate in APCI mode, (B) lutein-3’-*O*-palmitate in APCI mode. *major non-carotenoid peaks

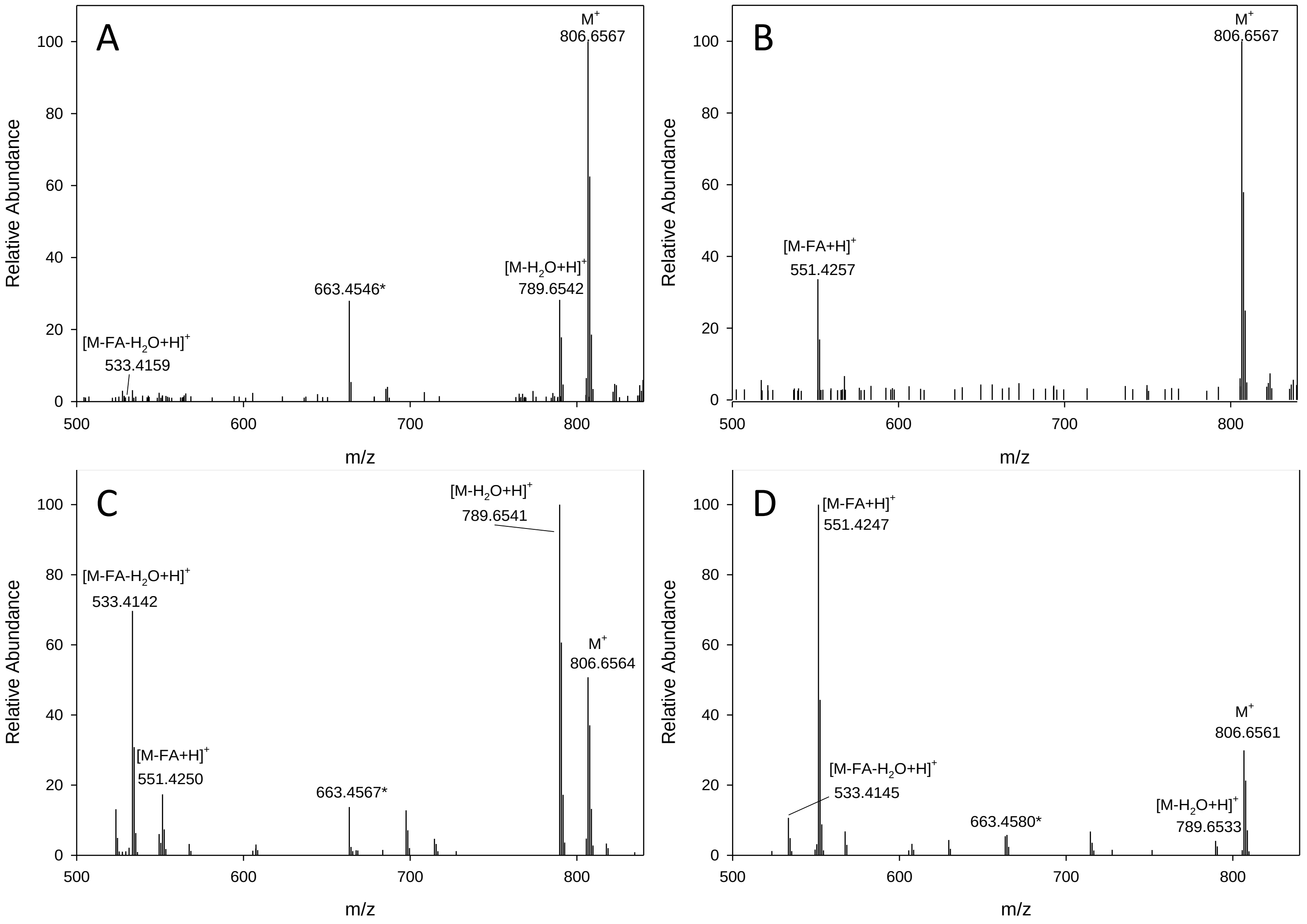

Supplementary Table S1

Quality and nutrient content of muffin products

Attribute	Hairless canary seed (100%)	Hairless canary seed/corn blend (1:1, w/w)	Hairless canary seed/corn blend (1:2, w/w)
Height (cm)	3.5 ±0.09	3.3 ±0.07	3.0 ±0.09
Internal quality score (5-point scale)	3.6 ±0.13	3.9 ±0.17	4.1 ±0.18
External quality score (5-point scale)	4.6 ±0.12	4.7 ±0.14	4.9 ±0.14
Moisture (%, wb)	27.5 ±0.57	28.5 ±0.35	28.9 ±0.53
Protein (%, db)	17.9 ±0.12	11.0 ±0.49	9.2 ±0.16
TDF (%, db)	12.8 ±0.63	8.4 ±0.38	7.8 ±0.12
Fat (%, db)	5.7 ±0.21	4.5 ±0.21	3.8 ±0.15
Ash (%, db)	3.3 ±0.05	2.5 ±0.06	2.3 ±0.05

Supplementary Table S2

Quality and nutrient content of bread products

Attribute	Wheat (100%)	Wheat/Hairless canary seed (85/15, w/w)	Wheat/Hairless canary seed (75/25, w/w)	Wheat/Hairless canary seed (50/50, w/w)
Loaf volume (cm^3)	455 ±19	428 ±15	395 ±12	308 ±11
Internal quality score (10-point scale)	7.2 ±0.28	6.3 ±0.21	6.2 ±0.22	4.0 ±0.18
External quality score (10-point scale)	7.5 ±0.25	6.9 ±0.19	6.8 ±0.19	4.1 ±0.17
Moisture (%, wb)	35.1 ±0.76	35.5 ±0.62	36.3 ±0.97	35.0 ±1.03
Protein (%, db)	18.4 ±0.44	20.4 ±0.31	20.6 ±0.55	21.7 ±0.69
TDF (%, db)	11.5 ±0.39	11.9 ±0.53	12.3 ±0.49	12.6 ±0.47
Fat (%, db)	2.5 ±0.09	2.8 ±0.10	3.6 ±0.15	4.2 ±0.13
Ash (%, db)	3.2 ±0.08	3.3 ±0.06	3.4 ±0.07	3.5 ±0.09
